# Variability in radiotherapy outcomes across cancer types: a comparative study of glioblastoma multiforme and low-grade gliomas

**DOI:** 10.18632/aging.206212

**Published:** 2025-02-27

**Authors:** Alexander Veviorskiy, Garik V. Mkrtchyan, Andreyan N. Osipov, Evgeny Izumchenko, Ivan V. Ozerov, Alex Aliper, Alex Zhavoronkov, Morten Scheibye-Knudsen

**Affiliations:** 1 Insilico Medicine AI Limited, Abu Dhabi, UAE; 2 Department of Cellular and Molecular Medicine, Center for Healthy Aging, University of Copenhagen, Denmark; 3 CANDLE Synchrotron Research Institute, Yerevan 0040, Armenia; 4 Department of Medicine, Section of Hematology and Oncology, University of Chicago, Chicago, IL 60637, USA; 5 Insilico Medicine Hong Kong Limited, Hong Kong Science and Technology Park, Hong Kong; 6 Insilico Medicine Canada Inc., René-Lévesque Blvd W, Montreal, Quebec H3B 4W8, Canada; 7 Buck Institute for Research on Aging, Novato, CA 94945, USA

**Keywords:** cancer, biomarkers, radiotherapy, GBM, LGG, survival

## Abstract

Radiotherapy is a crucial treatment option for various cancers. However, the results of radiotherapy can vary widely across different cancer types and even among patients with the same type of cancer. This variability presents a major challenge in optimizing treatment strategies and improving patient survival. Here, we collected radiotherapy phenotype and expression data from 32 TCGA cancer datasets and performed overall survival analysis for 32 cancer types. Additionally, we conducted a signaling pathway enrichment analysis to identify key pathways involved in radiotherapy resistance and sensitivity. Our findings show that radiotherapy improves survival outcomes in certain cancer types, such as glioblasoma multiforme (GBM), while worsening outcomes in others, such as low-grade glioma (LGG). Next, we focused on exploring the differences in radiotherapy outcomes between GBM and LGG, focusing on the molecular mechanisms contributing to these variations. We identify differential regulation of pathways related to programmed cell death, DNA repair, telomere maintenance, chromosome condensation, antiviral responses, and interferon signaling between GBM and LGG patients perhaps explaining radiotherapy efficacy. A genetic analysis confirmed the importance of immune response and radiotherapy outcome for LGG patients. These insights underscore the importance of personalized treatment approaches and the need for further research to improve radiotherapy outcomes in cancer patients.

## INTRODUCTION

Radiotherapy plays a fundamental role in the treatment of cancer. Even though radiotherapy is widely used, its outcomes can vary significantly depending on the cancer type. It was observed that GBM patients who received radiotherapy have better overall survival than those who did not receive radiotherapy [1]. Conversely, it was observed to have the opposite effect with LGG patients [2]. Tumor heterogeneity is a major factor that affects radiotherapy response rates, even among patients diagnosed with the same tumor type [3]. The variability in radiotherapy outcomes across cancer types may lie in the complex interactions between treatment, anatomical, tumor and patient-related variables. These interactions can significantly influence treatment efficacy and patient prognosis [4]. Additionally, the use of radiotherapy varies significantly across different cancer diagnoses, and understanding these variations can help improve treatment strategies [5]. GBM and LGG are particularly interesting to study together because GBM often originates from a preexisting LGG, representing a progression from a lower-grade to a higher-grade malignancy [6]. This progression is associated with significant changes in gene expression profiles [7], which may underlie the differences in radiotherapy outcomes observed between these two cancer types. Understanding these differences is crucial, as it can inform personalized treatment strategies and improve survival outcomes.

Recent advancements in radiotherapy for GBM and LGG include the exploration of various targeted therapies [8], novel radiotherapy approaches, and immunotherapies. For instance, vaccine strategies have shown promising results in early-phase clinical studies [9]. Particle irradiation and dose escalation strategies, including modern molecular imaging, are being evaluated for their long-term outcomes [10]. Intraoperative radiotherapy (IORT) is being explored to sterilize the margins from persistent tumor cells and bridge the therapeutic gap between surgery and radio chemotherapy [11]. Immunotherapy is another promising modality, with radiotherapy potentially enhancing the effect of immunotherapy through various mechanisms. These include understanding the microenvironment to overcome “tumor coldness” and employing dual immunotherapy, which targets immune pathways at different stages and through different receptors [12]. The combination of radiotherapy with targeted therapies against key DNA damage response players, including TP53, WEE1, BRCA, and PARP, is another example of promising combinations, as DNA damage is a key mechanism by which tumor cell death might be achieved [13]. PARP inhibitors have been shown to sensitize high-grade glioma, medulloblastoma, and ependymoma cell lines to ionizing radiation [14].

Despite these advancements, the survival rate for GBM patients has not significantly improved in recent years, indicating a need for novel therapies that could be used in conjunction with standard radio chemotherapy approaches [15]. Radiotherapy resistance is frequently observed in GBM patients and is a major cause of the high mortality rate [16]. This resistance is often multifactorial and heterogeneous, associated with the recurrence of GBM after surgery [17]. Similarly, advancements in radiotherapy for LGG have been significant, driven by improvements in neuroimaging, radiotherapy planning, and delivery techniques, contributing to better tumor control and reduced radiation-related toxicity [18]. Despite these advancements, the role of radiotherapy in LGG remains debated, with some studies failing to demonstrate a radiotherapeutic dose-response effect [19]. The optimal timing of radiotherapy is also debated, with some evidence suggesting that delayed and reduced dose irradiation may be beneficial [20].

In response to these challenges, our study aims to investigate the variability in radiotherapy outcomes between GBM and LGG patients. We collected radiotherapy phenotype data for 32 TCGA cancer datasets and performed an overall survival analysis for 32 cancer types. We also conducted a signaling pathway enrichment analysis to uncover the underlying biological processes that may contribute to the observed differences in radiotherapy outcomes for GBM and LGG cancer patients. A differential regulation of pathways related to programmed cell death, DNA repair, telomere maintenance, chromosome condensation, antiviral responses, and interferon signaling was observed. Importance of the immune response in radiotherapy outcome for LGG cancer patients were confirmed using genetic data. The findings could have a significant impact on personalized treatment approaches and novel co-treatment approaches with radiotherapy.

## MATERIALS AND METHODS

### Data collection and differential expression analysis

Gene expression, phenotype, and survival data for 32 TCGA cancers and CNV data (GDC GISTIC copy number dataset) for TCGA-LGG and TCGA-GBM cancers and mutation data (MuTect2 Variant Aggregation and Masking) for TCGA-LGG cancers were collected from the UCSC Xena database [21]. CNV and mutation data were already preprocessed according to the NCI GDC pipeline. Definitions of all available in TGCA data mutations were manually categorised into 4 major groups including “protein coding”, “disruptive protein coding”, “splice site” and “noncoding” (Supplementary Table 1, Column called “All detected mutations in TCGA cancers” describes the original name of mutations provided in UCSC Xena database, columns called “category” represents the names of the mutations categorized into 4 major groups). FPKM-UQ expression data obtained from patients’ samples were uploaded into PandaOmics [22] and preprocessed according to the PandaOmics pipeline. Differential expression analysis was performed using the limma package for TCGA-GBM and TCGA-LGG datasets, comparing patients who received radiotherapy to those who did not receive radiotherapy. The obtained gene-wise *p*-values were corrected using the Benjamini–Hochberg procedure. Differential expression results were later used for gene set enrichment analysis.

### Overall survival analysis of IR-treated and IR-untreated cancer patients

Survival analysis was conducted using the KaplanMeierFitter function from the lifelines Python package. Patients were divided into two groups: those who received radiotherapy and those who did not receive radiotherapy. Only patients with available expression data and known irradiation status were included in the analysis. Briefly, 32 TCGA cancers (Supplementary Table 2) were analyzed, and survival analysis was performed between the two described groups of patients. The log-rank test was used to calculate statistical significance. The significance of survival outcomes was plotted on a heatmap and colored red if radiotherapy increased survival outcomes and blue if the application of radiotherapy decreased survival outcomes. Non-significant results were colored white. Combined survival plots for patients who received and did not receive radiotherapy across all TCGA cancers were plotted using the matplotlib package. Sub-stratification of IR-treated GBM and LGG patients was performed according to CNV loss or gain status of a gene compared with neutral status of the same gene. Only genes that exhibit alterations in at least 10% of cancer-specific patient samples were considered for the survival analysis.

### Signaling pathway enrichment analysis

Pathway enrichment analysis was performed using the GSEApy package with the enrichr() function, following standard protocols. The Reactome database was selected as the source of gene sets from the GSEApy internal library for signaling pathway enrichment analysis. Genes that were significantly up-regulated in TCGA-GBM and simultaneously down-regulated in TCGA-LGG, and vice versa, were used as input for the pathway enrichment analysis. The top 20 significantly perturbed signaling pathways were visualized on a dot plot using the GSEApy.plot function. Additionally, genes whose loss of function compared with neutral status were associated with significant stratification of IR-treated LGG patients were used as input for the pathway enrichment analysis. The top 20 significantly perturbed signaling pathways were visualized on a dot plot using the GSEApy.plot function.

### Paper draft preparation

The draft of this paper was generated using DORA, Insilico Medicine’s LLM-based paper drafting assistant. Draft Outline Research Assistant (DORA) is designed to streamline the process of publication creation, making it faster and simpler. The process of paper generation is curated by over 30 AI agents, powered by Large Language Models (LLMs), and integrated with internal and other curated databases, to assist in generating high-quality scientific papers. Each agent employs Retrieval-Augmented Generation (RAG) to perform comprehensive data collection and analysis, reduce the probability of hallucinations, and provide relevant PubMed links to make the generation of the paper more transparent. Followed by generation, the draft was manually curated and extended by the authors.

### Data availability

All data supporting the conclusions of the paper are available in the article and corresponding figures. TCGA datasets used in the paper are described in the materials and methods section.

## RESULTS

### Patient and tumor characteristics in TCGA cancers

**Figure 1:**
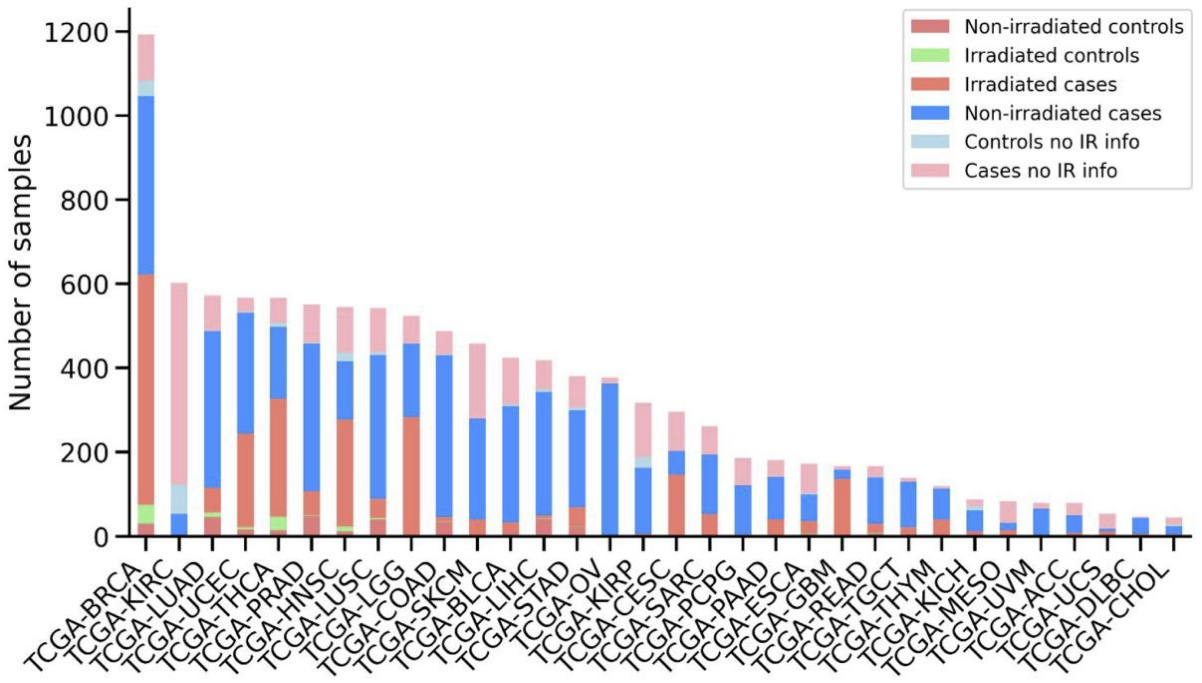
**Overview of the patient’s samples downloaded from TCGA.** Number of samples for each cancer type is shown, sorted by the total number of samples. Stacked bars are colored according to the sample category, including non-irradiated control samples, irradiated control samples, irradiated case samples, non-irradiated case samples, control samples without information about radiotherapy and case samples without information about radiotherapy.

Radiotherapy phenotype data were collected for 32 TCGA cancer datasets from the UCSC Xena database. TCGA-LAML was excluded from the analysis since there were no patients who received radiotherapy. The total number of samples varies between 1,194 and 45 for TCGA-BRCA and TCGA-CHOL, respectively. Similarly, the percentage of patients who received radiotherapy varies between 82% and 0% for TCGA-GBM and TCGA-KIRC/TCGA-CHOL/TCGA-KICH/TCGA-KIRP, respectively. It was noted that TCGA-GBM and TCGA-LGG are the cancers with the highest percentage of patients who received radiotherapy, at 82% and 54%, respectively (Figure 1).

### Overall survival analysis of patients who received and did not receive radiotherapy

**Figure 2:**
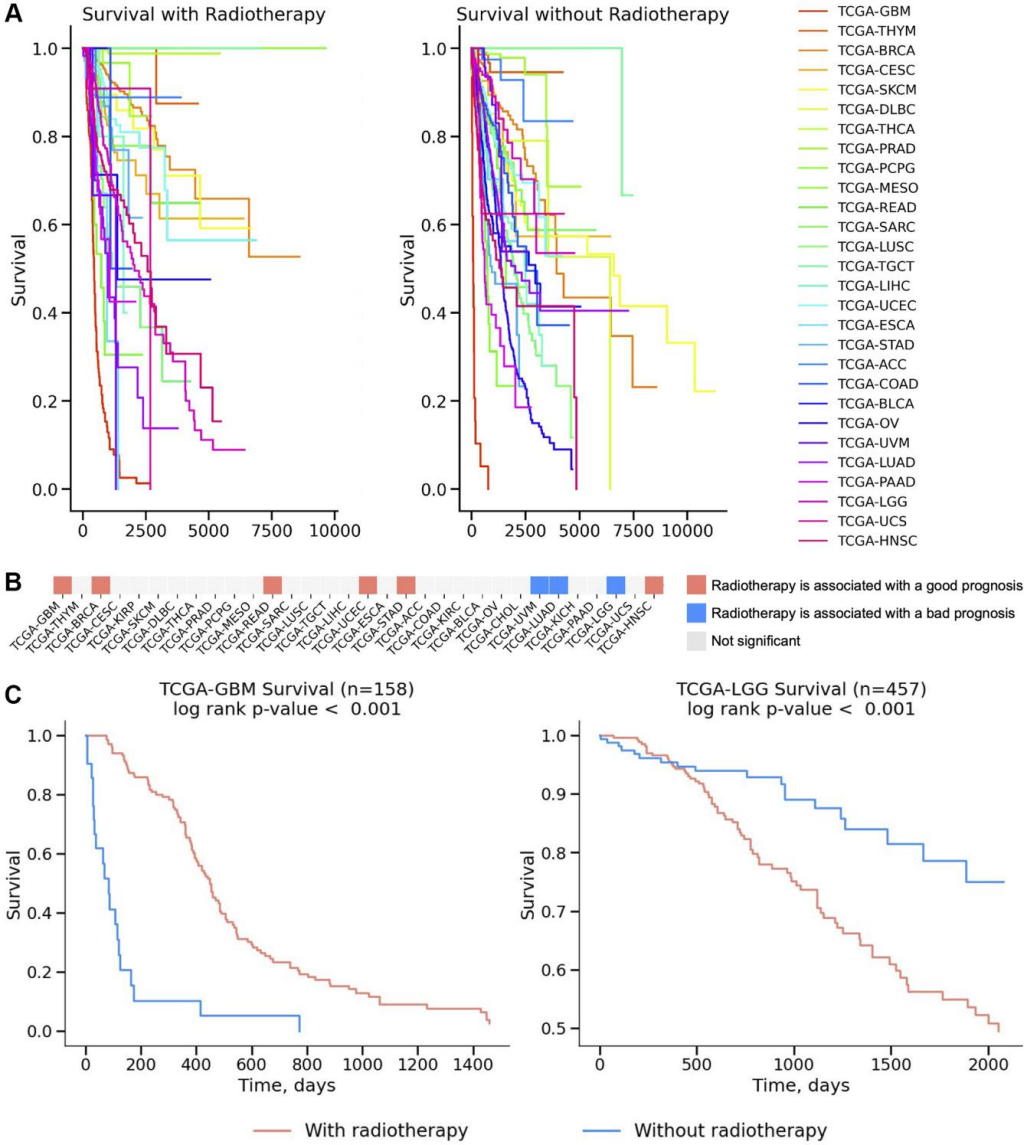
**Survival analysis across 32 TCGA cancers.** (**A**) Survival curves for patients who received radiotherapy and those who didn’t presented on a Kaplan-Meier plot for 32 TCGA cancers. (**B**) The significance of survival results is plotted on a heatmap and colored red if radiotherapy increased survival outcomes and blue if the application of radiotherapy decreased survival outcomes. Non-significant results were colored white (*p*-value > 0.05). The log-rank test was used to calculate statistical significance. (**C**) Survival analysis for IR treatment is presented on a Kaplan–Meier plot for TCGA-GBM (Glioblastoma, left figure) and TCGA-LGG (Brain Lower Grade Glioma, right figure) cancers.

To study whether radiotherapy treatment could be used as a trait capable of stratifying cancer patients with different outcomes, we performed an overall survival analysis for 32 cancer types from the TCGA dataset. The overall survival analysis was conducted for patients who received radiotherapy and for those who did not receive radiotherapy (Figure 2A). In some cases, radiotherapy can improve survival outcomes, while in others, it worsens them. For example, it was found that patients with GBM, BRCA, READ, UCEC, STAD, and HNSC who received radiotherapy lived longer compared to patients who did not receive radiotherapy. Conversely, it was noted that patients with UVM, LUAD, and LGG who received radiotherapy had shorter survival compared to those who did not receive radiotherapy (Figure 2B). This observation led us to focus on the differences between GBM (Figure 2C, left) and LGG (Figure 2C, right) patients who received and did not receive radiotherapy, since GBM can develop from LGG.

### Signaling pathway enrichment analysis of GBM and LGG patients and patients’ genetic profiles

**Figure 3:**
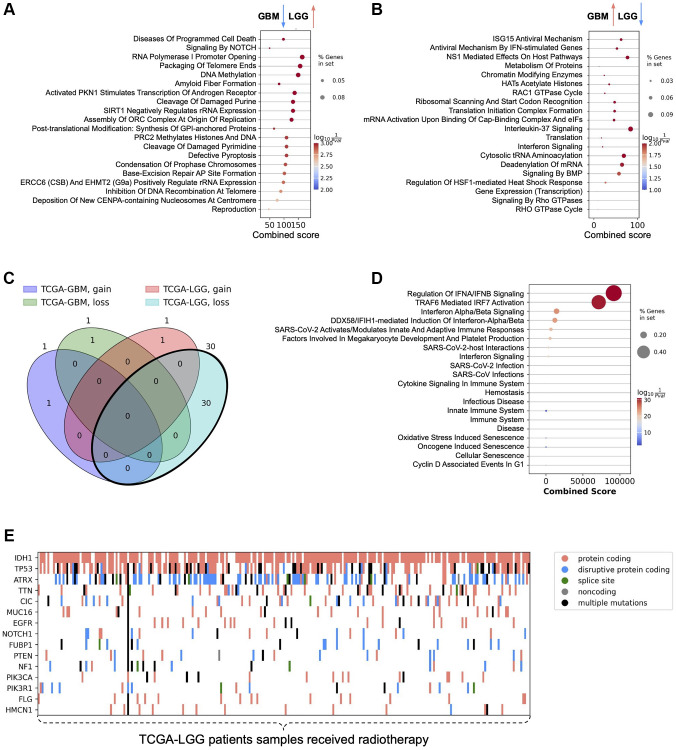
**Signaling pathway enrichment analysis results and patients' genetic profiles.** (**A**) Signaling pathways enriched with genes significantly down-regulated in the comparison of IR-treated and untreated GBM patients and simultaneously significantly up-regulated in the comparison of IR-treated and untreated LGG patients. (**B**) Signaling pathways enriched with genes significantly up-regulated in the comparison of IR-treated and untreated GBM patients and simultaneously significantly down-regulated in the comparison of IR-treated and untreated LGG patients. (**C**) Venn diagram describing the intersection of genes whose loss/gain status significantly stratifies IR-treated patients. (**D**) Signaling pathways enriched with genes whose loss status in TCGA-LGG IR-treated patients is associated with worse prognosis. (**E**) Mutation profile of low-grade glioma cancer tissues in patients receiving radiotherapy.

To find differences at the transcriptional level between GBM and LGG patients who were exposed to radiotherapy and those who were not, we collected gene expression data for those patients. Then we created comparisons between patients who received radiotherapy and those who did not, and calculated differentially expressed genes. After that, we obtained a list of genes that were significantly down-regulated in GBM patients and at the same time significantly up-regulated in LGG patients, and vice versa. Those gene lists were used for pathway enrichment analysis to uncover the underlying biological processes contributing to the observed differences in radiotherapy outcomes (Figure 3). The rationale behind the pathway enrichment analysis choices was to identify and understand the specific biological processes and molecular pathways that are differentially regulated in response to radiotherapy in GBM and LGG patients, thereby providing insights into the mechanisms underlying their distinct responses to treatment and potentially revealing targets for therapeutic intervention. It was noted that pathways related to programmed cell death and DNA repair, such as “Diseases Of Programmed Cell Death” and “Base-Excision Repair,” were down-regulated in GBM but up-regulated in LGG. This suggests a potential mechanism by which LGG cells might be more susceptible to radiotherapy-induced damage, whereas GBM cells might evade such damage. It was also observed that pathways involved in telomere maintenance and chromosome condensation, such as “Packaging Of Telomere Ends” and “Condensation Of Prophase Chromosomes,” were differentially regulated, indicating a possible role in the differential radiotherapy outcomes between GBM and LGG (Figure 3A). Moreover, pathways associated with antiviral responses and interferon signaling, including “ISG15 Antiviral Mechanism” and “Interferon Signaling,” were up-regulated in GBM and down-regulated in LGG. This could imply an enhanced immune response in GBM, potentially contributing to its resistance to radiotherapy. Finally, pathways related to gene expression and protein metabolism, such as “Gene Expression (Transcription)” and “Metabolism Of Proteins,” were up-regulated in GBM and down-regulated in LGG, suggesting a higher metabolic and transcriptional activity in GBM that might support its aggressive nature and resistance to treatment (Figure 3B).

Next, we focused on sub-stratification of GBM and LGG patients who were exposed to IR, according to CNV status of genes that have genetic perturbation (loss or gain of function) in at least 10% of cancer-specific patient samples. It was noted that both genetic gain and loss abnormalities are associated with worsening survival outcomes of patients who received radiotherapy (Figure 3C). Particularly, gain of function of KIT and loss of function of SPTBN5 genes compared with corresponding neutral CNV statuses of KIT and SPTBN5 genes respectively are associated with worse prognosis of IR-treated GBM patients. Survival analysis of IR-treated LGG patients revealed that there is only one gene whose gain of function significantly worsens survival outcome - EGFR. At the same time, loss of function of 30 genes, including CDKN2B, CDKN2A, MTAP, ELAVL2, IZUMO3, DMRTA1, IFNA8, IFNE, IFNA1, IFNA2, KLHL9, IFNA13, IFNA5, IFNA6, IFNW1, IFNA21, IFNA14, IFNA4, IFNA17, IFNA10, IFNA7, IFNA16, FOCAD, IFNB1, HACD4, MLLT3, NGEF, GBX2, ASB18, and AGAP, is associated with worse prognosis in IR-treated LGG patients. Signaling pathway enrichment analysis of these genes revealed that the major pathways are related to immune response and cellular processes, including “Interferon Signaling” and other immune response and cellular processes (Figure 3D). These results are in agreement with the previously obtained results on the gene expression level, suggesting that immune modulation of LGG patients is important for survival outcomes.

Finally, the mutation profile of LGG patient samples that received radiotherapy was obtained. The top 15 genes with the highest number of mutations across radiotherapy-received LGG patients are plotted on a heatmap (Figure 3E). Among the top 15 genes, IDH1 and TP53 stand out due to their well-known roles in glioma biology. ATRX was found to be the gene with the highest number of disruptive protein-coding mutations. Interestingly, EGFR was also observed in the list of the top 15 most mutated genes across IR-treated LGG patients.

## DISCUSSION

The study presented here shows the variability in radiotherapy outcomes across different cancer types, with a specific focus on glioblastoma multiforme (GBM) and low-grade gliomas (LGG). One of the unique aspects of this study is its focus on the differential radiotherapy outcomes between GBM and LGG. While radiotherapy remains a fundamental treatment for GBM, the high resistance to treatment and subsequent poor prognosis emphasize the need for novel therapeutic strategies [23]. Conversely, LGG patients often show a more favorable response to radiotherapy, which can be predicted by MRI evaluations post-treatment [24]. The findings from this study have the potential to impact clinical practices and treatment protocols by providing a more detailed understanding of how radiotherapy should be tailored to individual patients based on their specific cancer type.

Radiotherapy was found to improve survival outcomes in GBM while worsening outcomes in LGG. GBM and LGG, despite their differences, share a common lineage, with GBM often developing from LGG [6]. The differential regulation of pathways related to programmed cell death, DNA repair, telomere maintenance, chromosome condensation, antiviral responses, and interferon signaling between GBM and LGG patients those receiving radiotherapy or not may explain the underlying reasons for these observed differences. For instance, the down-regulation of DNA repair pathways in GBM suggests a mechanism for radiotherapy resistance, while their up-regulation in LGG indicates a higher susceptibility to radiotherapy-induced damage. The use of radiotherapy in combination with temozolomide has been shown to improve survival rates in GBM patients [25]. However, radiotherapy resistance remains a significant challenge, often leading to poor outcomes [16]. In LGG, the timing and dosage of radiotherapy are crucial factors that can influence survival outcomes [26]. Given the up-regulation of DNA repair pathways in LGG, combining radiotherapy with DNA repair inhibitors could make LGG cells more vulnerable to treatment. For example, using PARP inhibitors could enhance the effectiveness of radiotherapy [27]. On the other hand, the down-regulation of antiviral and interferon signaling pathways in LGG indicates a less active immune environment. Taking into account additional confirmation of the importance of the immune response at the genetic level, combining radiotherapy with immune modulators, such as interferon therapy or immune checkpoint inhibitors, could boost the immune response against LGG cells and improve treatment outcomes [28]. It was also found that ATRX loss of function is associated with increased radiosensitivity in GBM [29, 30]. Furthermore, LGG patients who received radiation therapy and carried disruptive protein-coding mutations in the ATRX gene had prolonged survival compared to radiotherapy-treated patients without disruptive protein-coding mutations in the ATRX gene (Supplementary Figure 1). This suggests that the status of ATRX might be used as a biomarker for both GBM and LGG. EGFR was previously associated with radioresistance in various cancers, including oropharyngeal carcinoma, head and neck cancer, and GBM [31–33]. Identifying the link between the amplification of the EGFR gene in LGG patients and the worsening survival prognosis of radiotherapy-treated patients might be beneficial for patient stratification before therapy assignment. The results of this study align with these findings, further emphasizing the importance of personalized treatment approaches. While this study provides valuable insights into the differential radiotherapy outcomes between GBM and LGG, it is important to acknowledge potential limitations associated with our methodology. Specifically, biases may arise from dataset heterogeneity, as variations in patient demographics, treatment regimens, and data collection methods could influence the observed results and interpretations.

In conclusion, this study provides valuable insights into the variability in radiotherapy outcomes across different cancer types, with a specific focus on GBM and LGG. The identification of key pathways involved in radiotherapy resistance and sensitivity offers potential biomarkers for future therapeutic strategies. The findings highlight the importance of personalized treatment approaches and further research into the molecular mechanisms behind radiotherapy response along with the development of novel therapeutic strategies to improve clinical outcomes for patients with these distinct types of brain tumors.

## Supplementary Materials

Supplementary Figure 1

Supplementary Tables
