# Supplementary material for: Variability in radiotherapy outcomes across cancer types: a comparative study of glioblastoma multiforme and low-grade gliomas
Source: Aging (Albany NY). 2025 Feb 27;17(2):550–62. doi: 10.18632/aging.206212 (PMC11892922; doi:10.18632/aging.206212)
Supplement: Supplementary Figure 1 [file aging-17-206212-s001.pdf]

SUPPLEMENTARY FIGURE

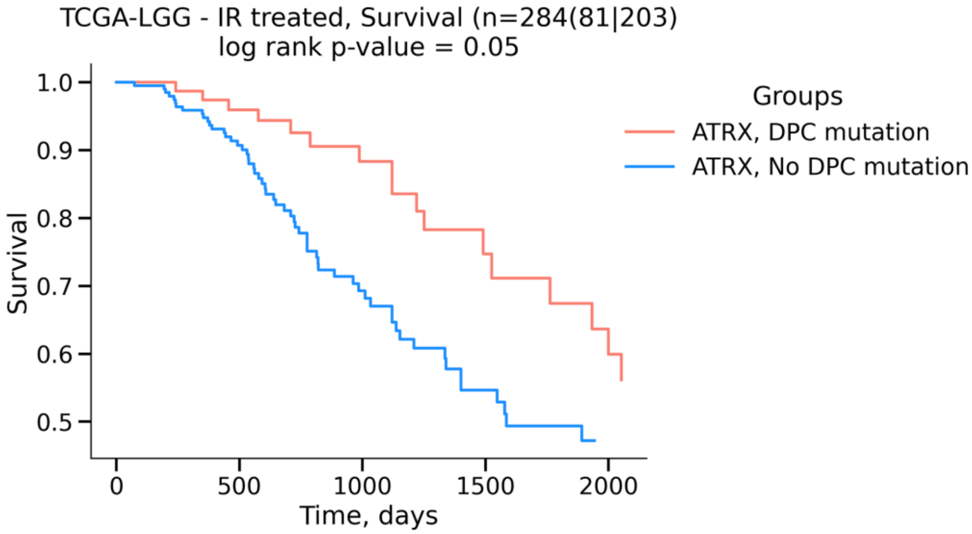

Supplementary Figure 1. Survival analysis for the group of LGG patients who received radiation therapy and carried disruptive protein-coding mutations in the ATRX gene compared to radiotherapy-treated patients without disruptive protein-coding mutations in the ATRX gene.
