# Supplementary material for: Variability in radiotherapy outcomes across cancer types: a comparative study of glioblastoma multiforme and low-grade gliomas
Source: Aging (Albany NY). 2025 Feb 27;17(2):550–62. doi: 10.18632/aging.206212 (PMC11892922; doi:10.18632/aging.206212)
Supplement: Supplementary Tables [file aging-17-206212-s002.pdf]

SUPPLEMENTARY TABLES

Supplementary Table 1. Original names of the mutations and their categories in all available TCGA data mutation types.

All detected mutations in TCGA cancers	Category
missense_variant	Protein coding
splice_acceptor_variant	Splice site
intron_variant	Splice site
frameshift_variant	Disruptive protein coding
mature_miRNA_variant	Noncoding
stop_lost	Disruptive protein coding
downstream_gene_variant	Noncoding
regulatory_region_variant	Noncoding
splice_region_variant	Splice site
non_coding_transcript_exon_variant	Splice site
intergenic_variant	Noncoding
start_lost	Disruptive protein coding
splice_donor_variant	Splice site
coding_sequence_variant	Protein coding
non_coding_transcript_variant	Noncoding
stop_retained_variant	Protein coding
protein_altering_variant	Protein coding
NMD_transcript_variant	Disruptive protein coding
upstream_gene_variant	Noncoding
incomplete_terminal_codon_variant	Protein coding
inframe_insertion	Disruptive protein coding
inframe_deletion	Disruptive protein coding
synonymous_variant	Protein coding
3_prime_UTR_variant	Noncoding
5_prime_UTR_variant	Noncoding
stop_gained	Disruptive protein coding

Supplementary Table 2. TCGA cancer abbreviations and their full names.

TCGA abbreviation	Cancer
TCGA-GBM	Glioblastoma multiforme
TCGA-THYM	Thymoma
TCGA-BRCA	Breast invasive carcinoma
TCGA-CESC	Cervical squamous cell carcinoma and endocervical adenocarcinoma
TCGA-KIRP	Kidney renal papillary cell carcinoma
TCGA-SKCM	Skin Cutaneous Melanoma
TCGA-DLBC	Lymphoid Neoplasm Diffuse Large B-cell Lymphoma
TCGA-THCA	Thyroid carcinoma
TCGA-PRAD	Prostate adenocarcinoma
TCGA-PCPG	Pheochromocytoma and Paraganglioma
TCGA-MESO	Mesothelioma
TCGA-READ	Rectum adenocarcinoma
TCGA-SARC	Sarcoma

TCGA-LUSC	Lung squamous cell carcinoma
TCGA-TGCT	Testicular Germ Cell Tumors
TCGA-LIHC	Liver hepatocellular carcinoma
TCGA-UCEC	Uterine Corpus Endometrial Carcinoma
TCGA-ESCA	Esophageal carcinoma
TCGA-STAD	Stomach adenocarcinoma
TCGA-ACC	Adrenocortical carcinoma
TCGA-COAD	Colon adenocarcinoma
TCGA-KIRC	Kidney renal clear cell carcinoma
TCGA-BLCA	Bladder Urothelial Carcinoma
TCGA-OV	Ovarian serous cystadenocarcinoma
TCGA-CHOL	Cholangio carcinoma
TCGA-UVM	Uveal Melanoma
TCGA-LUAD	Lung adenocarcinoma
TCGA-KICH	Kidney Chromophobe
TCGA-PAAD	Pancreatic adenocarcinoma
TCGA-LGG	Brain Lower Grade Glioma
TCGA-UCS	Uterine Carcinosarcoma
TCGA-HNSC	Head and Neck squamous cell carcinoma
